# Application of OECD LSE Framework to Assess Spatial Differences in Rural Green Development in the Arid Shaanxi Province, China

**DOI:** 10.3390/ijerph17010286

**Published:** 2019-12-31

**Authors:** Boyang Zhou, Wenxin Liu, Weinan Lu, Minjuan Zhao, Linfei Li

**Affiliations:** College of Economics and Management, Northwest A&F University, Yangling 712100, China; amado1988@163.com (B.Z.); liuwenxin@nwafu.edu.cn (W.L.); luweinan@nwafu.edu.cn (W.L.); linfei_0912@163.com (L.L.)

**Keywords:** green development, MCDM methods, LSE model, arid rural China, spatial analysis

## Abstract

The green development theory proposed by the Organization for Economic Cooperation and Development (OECD) has promoted the harmonious development of the economy, society, and environment in many countries, in particular, it has provided a good option for the coordinative development of economic growth, resource utilization, and ecological protection in rural areas of developing countries. For this reason, we used the OECD model to measure green development in arid, rural areas of China, and also subjective and objective weighting methods to measure the rural green development level of 78 county-level regions in Shaanxi province in 2018. At the same time, the least square error (LSE) method was used to determine the contribution rate of government support, environmental pressure, resource endowment, and quality of life, so as to determine the influencing factors of rural green development in Shaanxi. The results show that the levels of rural green development in Shaanxi province differed internally: the level of green development in the north was strong, moderate in the southwest and northwest, and weak in the center and south. The driving types of rural green development in Shaanxi province are divided into five types: Three Factors I, Three Factors II, Four Factors I, Four Factors II, and Five Factors; the influencing factors of rural green development are varied from county to county. In terms of different regions, different development approaches and countermeasures are proposed respectively. This research provides scientific guidance for local government to formulate green agricultural development policies and to overcome the development difficulties in rural areas.

## 1. Introduction

Since 1949, the Chinese government has focused on rural development and striving for the harmonious development of rural society, the economy, and the environment. Since the economic reform and opening-up, China’s rural economy has rapidly developed and achieved remarkable progress. Comparing 1978 to 2018, grain output and rural residents’ income had increased by 2.17 and 110 times, respectively. During this rapid development, high energy consumption, and a high emissions rate have placed enormous pressure on rural resources and the environment. Agricultural diffuse source pollution, land desertification, water shortage, and other widespread issues have highlighted the importance of sustainable rural development [1,2,3]. In response to this problem, in 2018, the central committee of the Communist Party of China incorporated the concept of green development into the planning of new rural construction which emphasized that environmental capacity and ecological carrying capacity should be considered during development and that the harmonious coexistence of human society and the ecological environment should be continuously promoted through the efficient use of resources and an appropriate level of economic development.

Organically combining the development, utilization, and management of resources, as well as the ability, rights, and livelihood benefits of people to use various resources, the philosophy of green development, extends the solution to sustainability issues of resources and the environment from the field of ecological environment to the fields of human development and social economy. In this way, a more comprehensive analysis of environmental protection issues can be performed, thereby a theoretical basis for resource utilization and management can be supplied, as well as opening a new path for the sustainable development of human society and the economy [4,5]. 

The research on green development was originated from the “Green Economy”, firstly proposed by the British environmental economist David Pearce in 1989. The “Green Economy” is conducive to transforming environmental protection behaviors into productivity, achieving sustainable economic development, and exploring the path to achieve sustainable development from the perspective of an environmental economy [6]. On this basis, Goodstein, Barnes, and other scholars formulated the green growth theory, emphasizing economic growth and assessing the contribution of natural assets to human welfare [7], which has been widely recognized. Subsequently, based on the green growth theory, the Organisation for Economic Co-operation and Development (OECD), the United Nations Environment Programme (UNEP), and other international organizations and scholars have introduced concepts or evaluation systems of green development [8], the green economy [9], and the green index [10]. Some indicator systems can sufficiently describe the capacity of green-development level by considering production, resources, consumption, and policy; some indicator systems can illustrate the relationship between the green economy and sustainable development in consideration of economic transformation, resource efficiency, social progress, and human well-being; some indicator systems can reflect the relationship between ecological resources and socioeconomic growth through the study of environmental status and policies. Some scholars introduced a dynamics system to conduct studies of the potential impact of the green economy on the regional economy, society, and systems [11]. 

However, China’s green development is mainly relegated to domestic research literature [12]. More explanations of the connotations of green development, taking the economy, society, and the environment as endogenous variables of green development, were provided by Zhou [13], Hu [14], Li [5], and other scholars. The internal rule of development can, therefore, be analyzed and studied, and the internal relationship between society, economy, and the environment can be understood; therefore, resource exhaustion and environmental degradation caused by the unilateral pursuit of rapid development can be avoided from a systematic and comprehensive perspective. Scholars such as Sun [15], Zhong [16], Liu [17], Liu [18], and Guo [19] have respectively studied and evaluated the functional evolution of China’s green-development system, green welfare in Chinese provinces, the green-development level of the Shandong province and all its cities, the green development of cities above the prefecture-level in China, and the green-development level of counties in Ningxia. 

In terms of research methods, the green-development level and index of different regions were evaluated, and a great number of index systems were constructed through application of the Technique for Order of Preference by Similarity to Ideal Solution (TOPSIS )model [19], case analysis [11], comprehensive index [20], cluster analysis [21], Data Envelopment Analysis(DEA-BCC) and the Malmquist model [15], data envelopment [22] and the function model [23]. In addition, through comprehensive review of relevant literature at home and abroad, researches on agricultural green development is mainly focused on the macro and micro levels of rural environmental issues. First of all, the research of rural environmental issues at the macro level can be divided into two parts: the first part is to divide the current environmental realities facing the rural areas, such as non-point source pollution, land desertification, soil erosion, etc. into independent research objects for specific analysis, and put forward pertinent countermeasures and suggestions [24]; the other part is to conduct an in-depth analysis and a preliminary exploration of rural environmental governance models from the perspective of the overall form of rural ecological environment problems [25,26,27]. Next, the micro-level research is mainly about the environmental issues related to individual farmers, such as the study on the treatment of domestic sewage and garbage [28]. However, accompanied by the development of China’s economy and society as well as the acceleration of urbanization, rural environmental problems are becoming increasingly complicated. Therefore, the research is no longer just to explore the problem, but also to analyze and find solutions to solve the rural ecological environment problems, combining the public’s psychological perception, behavioral performance and other factors outside the environment [29], such as seeking legalized governance on the basis of the willingness to pay for the treatment of domestic waste and its influencing factors [30]. To sum up, former literates’ investigations have already been quite abundant with a high reference value, but there are still some problems to be considered in the development of green agriculture.

Therefore, from the literature, we found that further exploration is needed in the following areas. Firstly, the existing researches on China’s green development are concentrated mainly at the national or provincial levels and the majority of which is in the eastern region [16]. However, for the western regions, especially the western rural areas that are usually accompanied by the most backward economic development and fragile ecological environment, it is more urgent to discover an evaluation model suitable for rural green development [16,31,32,33]. This can not only enrich the existing research content but also provide decision-makers with different scales of policy recommendations and more comprehensive policy reference, as well as understand the real data and situation from which problems can be identified. For this reason, a logical main line of “Rural social economy–agricultural production efficiency–farmers’ living standards–rural natural conditions–rural government management” will be set up and used to perform analysis in our study.

Secondly, since the release of China’s Human Development Report in 2002 [34], studies have been lacking on the overall form, obstacle factors, driving factors, and spatial-distribution characteristics of the green-development level in Western China, especially in rural areas [35]. As an important indicator of sustainable rural development, the green-development level indicates the nature of the problem from a more comprehensive and systematic perspective. 

Thirdly, the OECD developed a green development index to assess the relative sustainability of countries and regions. The index covers not only socioeconomic and government support, but also environmental and quality of life factors. It provides a new understanding and a relatively unique research perspective for solving the problems of sustainable growth. Other research results provided a theoretical basis for the comprehensive management of sustainable development and the harmonious development of human and society through comprehensive management [36]. Finally, the introduction of the OECD least square error (LSE) model helps deepen our understanding of the following aspects of rural green development: the OECD green development framework provides a theoretical basis and reference values for the planning and design services of relevant public sectors in rural areas. It outlines better target areas for providers, improves the reliability of programs, and maximizes the benefits of policy implementation. The application of the LSE (least square error, the minimum variance method) model [37] can help rural areas in China to better understand the factors driving green development, form accurate development plans, and ensure the maximum protection and use of the natural ecological environment while sustaining social and economic development. Relevant agencies and planners can be provided with data, analytical tools, and new research ideas to help identify the measures that are most important for the development of the region’s population, society, economy, government, and natural environment. 

Considering this, to identify the factors that most affect green development in rural areas in China and explore solutions to the problems of rural development, on the basis of the OECD green framework model, we calculated the green-development level of 78 counties in Shaanxi in 2018 and obtained the evaluation scores for five dimensions: social economy, environmental pressure, resource endowment, quality of life, and government support. Based on the contribution rate, we analyzed the factors driving rural green-development in these areas with the help of the LSE model. The purpose of the research was to reveal the problems with and spatial-distribution characteristics of rural green development in Shaanxi and to provide a theoretical reference for the formulation of rural development strategies.

## 2. The Study Area

**Figure 1:**
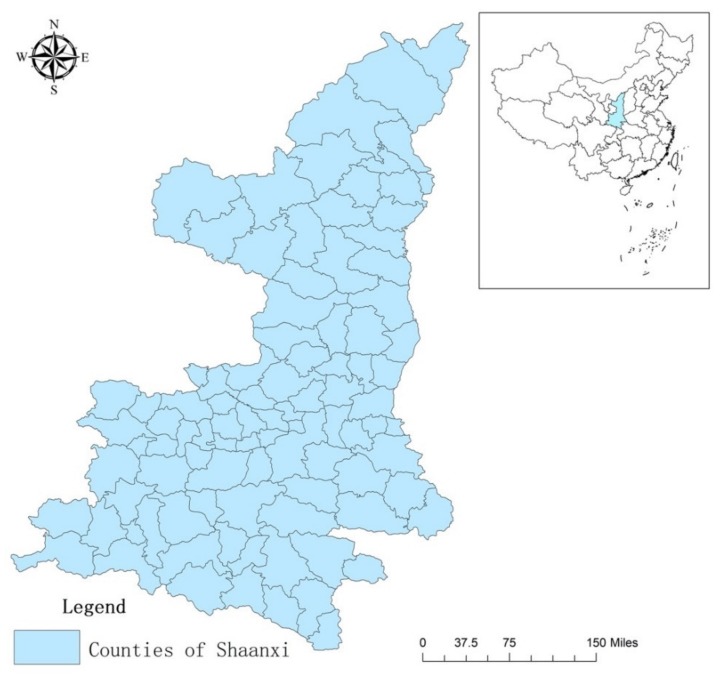
Study area in arid China.

Shaanxi province is located in the middle reaches of the Yellow River in the eastern part of Northwestern China, between 105°29′ and 111°15′ E and 31°42′ and 39°35′ N (Figure 1). It is a long and narrow province with high terrain in the north and south, and low terrain in the middle. From north to south, Shaanxi can be divided into three geomorphic regions: the northern Shaanxi plateau, the Guanzhong plain, and the Qinba mountain. The total land area is 205,600 km^2^, accounting for 2.145% of China’s total land area. Shaanxi currently has 107 counties, with Xi’an as its capital. We refer to rural areas of Shaanxi in a narrow sense, there are 78 counties in 10 prefecture-level cities, including one municipal district, six county-level cities, and 71 counties, accounting for 73% of the total number of administrative units in the whole province and composing the main development body of Shaanxi [38]. Since 1978, Shaanxi’s economic development has been rapid. The Grasso Domestic Product (GDP), calculated after adjusting for inflation, was 469.914 billion yuan in 2018, 57.96 times the 1978 value. The scale of agricultural production increased rapidly, and the total value of agriculture, forestry, animal husbandry, and fisheries exceeded 183.019 billion yuan in 2018, 50 times the 1978 value [39], the total output value of the province’s agriculture, forestry, animal husbandry, and fishery was 307.05 billion yuan, which is 84.6 times the value in 1978 [3]. The per capita disposable income of rural residents increased from 134 yuan in 1978 to 1265 yuan in 2017, a 9.4-fold increase [40]. However, the rural development of Shaanxi still faces many problems. (1) The gap between urban and rural income is still large. As of 2018, the ratio of urban and rural income residents in Shaanxi Province was 2.97:1, which is higher than the national average level (2.69:1). (2) The irrigation is seriously insufficient and the average effective irrigation rate (the ratio of effective irrigation to cultivated land area) in 2017 was only 31.6%, which is about 12% lower than the national average [41]. (3) About 60% of the whole province is experiencing soil erosion, which is one of the most serious instances of soil erosion in China [42]. (4) Due to ineffective management and development, the rural environment has deteriorated, and agricultural nonpoint-source pollution, domestic sewage discharge, and domestic waste treatment are serious issues [43]. In 2016, the annual output of garbage in rural areas of Shaanxi was 14.07 million tons, and the annual output of domestic sewage was about 386 million tons [44]. In terms of garbage treatment, 7732 administrative villages collecting garbage, accounting for 28.6% of the total number of administrative villages in Shaanxi, of which only 2495 administrative villages were treated, accounting for 9.24% of the total number of administrative villages in Shaanxi, far lower than the national average level. The main treatment method was to carry out simple treatment, which is a single landfill [3,5,45]. The number of township enterprises in Shaanxi has increased annually, but due to the restrictions of economic conditions and the management, emissions of sulfur dioxide, smoke, and wastewater have significantly increased, and some enterprises emitting serious pollution have exceeded the standard by 19.13 times [46]. In 2016, the chemical oxygen demand (COD) of agricultural emissions was 5176 tons, and the agricultural ammonia nitrogen emissions were 157 tons [44]. Excessive chemical-fertilizer and pesticide pollution have led to the deterioration of the rural ecological environment, which has considerably impacted the agricultural and rural development of Shaanxi. Therefore, to effectively improve the rural environment and realize sustainable development, conducting a comprehensive and systematic evaluation of the green development of rural areas in Shaanxi is necessary. 

## 3. Research Methods

### 3.1. OECD Green Development Model

The OECD provides a comprehensive set of indicators used to quantitatively evaluate the relative green development of countries or regions. The indicators reflect not only the basic situation of the region’s natural resources, but also management, human welfare, the environment, and economic growth and development [6,8]. The green-development evaluation index system constructed in this study was integrated from five dimensions: social economy, environmental pressure, resource endowment, quality of life, and government support. Several evaluation indices were included for each dimension. Based on the green development evaluation index system, an analytic hierarchy process (AHP) was applied to weigh the five-dimension systems to reflect the different adaptability to the objective situation of social resources having different adaptability to the scarcity of natural resources in different stages of social development. The formula is as follows:

(1) $$\mathrm{OECD}=\frac{\omega_{s}+\omega_{e}E+\omega_{r}R+\omega_{l}L+\omega_{g}G}{\omega_{s}+\omega_{e}+\omega_{r}+\omega_{l}+\omega_{g}}$$

where OECD represents the score matrix of green development evaluation: ω is s, e, r, l, or g, referring to the five dimensions of social economy, environmental pressure, resource endowment, quality of life, and government support, respectively. It refers to the weight of the corresponding subsystem obtained by the analytic hierarchy process. 

### 3.2. The LSE Model

The LSE (least square error, the minimum variance method) method is also known as the Weave composite index. With an increasing number of samples in a group of data, the variance first increases and then decreases to produce the minimum number of samples in the variance value, so it can reflect the actual situation in a region. According to Weave’s method, the ideal criterion for a single driving factor zone has only one dimension’s score accounting for all the total scores, with the scores of the other dimensions equaling zero. The significance of the two driving factors zone is that only the two dimensions score 50% for green development, while the other dimensions score 0. Similarly, only three dimensions of the three driving factors zone account for 33% of the total score of green development, and so on. According to Weave’s method, the composition of green development does not conform to any of the above theories. However, the real distribution can be compared with the theoretical distribution. The one with the smallest variance is closest to the theoretical distribution standard, so the real distribution can be regarded as identical to the theoretical one. According to the established standard, the contribution rate of the green development dimension after weighting a region was first calculated, and the contribution rates were ranked. Then, the variance formula was used to calculate variance when there were 1‒5 main driving factors. The dimension number with the smallest value was considered the main driver of green development. In this study, the OECD LSE model was introduced to conduct a spatial analysis of drivers of green development. Its formula is as follows:

(2) $$S^{2}=\frac{1}{n}\sum_{i=1}^{n}{\left(x_{i}-\;\bar{x}\right)}^{2}$$

where: $S^{2}$ represents variance;${\;x}_{i}$ represents sample data; $\bar{x}$ represents the average of samples; n represents the number of samples.

### 3.3. Index-System

According to the methods mentioned above, on the basis of the OECD evaluation system [8,9], combining ecological degradation, environmental pollution, resource depletion, human settlement degradation, and other problems faced by rural areas in Shaanxi province, and taking the actual development situation of rural areas in Shaanxi province as the starting point, we established an evaluation index system for rural green development in Shaanxi province in 2018, which was constructed from the five dimensions (Table 1).

#### 3.3.1. Social Economy (S)

The indicators of the social economy dimension reflect the current economic and social development of the region. The proportion of the added value of primary production in GDP (S1) refers to the ratio of the added value of the rural primary industry to rural GDP, which reflects the economic and industrial structure of rural areas [22]. The rural-urban income ratio (S2) refers to the ratio of per capita disposable income of rural-urban residents to per capita net income of rural residents, reflecting the prosperity of economic activities in rural areas [3]. Agricultural added value (S3) refers to the rural agricultural, forestry, husbandry, and fishery added value to the rural population, which is an indicator of rural productivity [22]. The urbanization rate (S4) refers to the ratio of the urban population to the rural population, reflecting the degree of rural modernization [22]. The financial self-sufficiency rate (S5) refers to the ratio of the general public budget to general public expenditure, which is a comprehensive indicator reflecting the financial strength of local governments [3]. Rural labor force transfer (S6) refers to the shift from agricultural production to secondary and tertiary industries for employment, reflecting the situation of rural labor productivity [21].

#### 3.3.2. Environmental Pressure (E)

The dimension of environmental pressure refers to a production model that can realize the efficient use of resources, environmental protection, economic growth, and sustainable and coordinated development, reflecting the production efficiency and sustainability of rural areas. The application intensity of pesticides and chemical fertilizers (E1) and the application intensity of agricultural plastic film (E2) refer to the ratio between the application amount of pesticides and chemical fertilizers and the cultivated land area, and the ratio between the amount of rural plastic film used and the cultivated land area, respectively [19]. In this paper, in a narrow sense, sewage treatment (E3) and the hazard-free treatment rate of waste (E5) refer to the agricultural wastewater amount and the ratio of the total sewage discharged and rural harmless garbage to the total quantity, respectively, reflecting the degree of the region’s sustainable development [15,19]. The total power of agricultural machinery per unit of cultivated land area (E4) refers to the ratio of the total power of agricultural machinery to rural cultivated land area, reflecting the degree of mechanization and production efficiency in rural areas [3].

#### 3.3.3. Quality of Life (L)

The indicators of the quality of life dimension refer to a series of objective and comprehensive indicators such as resident income, consumption level, living environment, sanitary conditions, and education level, which were analyzed and compared to reflect the improvement degree of regional economic, social, cultural, living, and other conditions. The rural nonhazardous toilet penetration rate (L1) refers to the ratio between the number of rural households using nonhazardous toilets and the total number of rural households, which is an indicator used to measure the quality of life of rural residents [15]. The per capita net income of rural residents (L2) refers to the total income of rural residents after deducting the expenses incurred when obtaining the income from various sources [15]. This indicator reflects the average income of rural residents in the region. The air quality index (L3) refers to the concentration of pollutants in the air and reflects the degree of air pollution [15]. Medical technicians per capita (L4) refers to the number of rural doctors and health technicians serving the rural population, which reflects the health situation in rural areas [15]. Total social consumer goods per capita (L5) refers to the total social consumer goods in rural areas compared to the rural population, which measures the level of rural consumption [15]. Grain yield per unit area (L6) refers to the ratio of total grain output to the sown area, which is an indicator of rural production efficiency [22].

#### 3.3.4. Government Support (G)

The dimension of government support reflects the management efficiency of the government in rural green development, which is mainly reflected in the management and support of the government via funds, policies, regulations, and other aspects affecting farmers’ lives, agricultural production, and the rural environment. The number of village committees per capita in rural areas (G1) refers to the number of village committees in rural areas compared to the rural population, which reflects the management degree of the rural government [8]. G2 refers to the ratio of rural expenditure on science and technology development to the total rural financial expenditure, which reflects the support for rural science and technology development [22]. Rural environmental protection (G3) refers to the ratio between the expenditure on environmental protection by the rural government and the total rural expenditure [21]. The proportion of rural agricultural and forestry water supplies (G4) is the ratio between the expenditure of the rural government on agricultural and forestry water supplies and the total rural expenditure, which reflects the development of rural agricultural and forestry water supplies [8]. The number of leading agricultural enterprises at the provincial level (G5) refers to the enterprises mainly engaged in the production, processing, and sales of agricultural products, which have reached the prescribed standards in terms of scale and operation indicators and have been recognized by the relevant government departments [8]. Per capita expenditure on education (G6) is the expenditure on education by rural governments compared to the rural population, which reflects the development degree of rural education [3].

#### 3.3.5. Resource Endowment (R)

The resource endowment dimension reflects the existing resource situation in rural areas. Rural cultivated land area per capita (R1) refers to the rural cultivated land area compared to the rural population, which indicates rural cultivated land resources [3]. Water resources per capita (R2) refers to the ratio between the total amount of water resources contained in springs, wells, rivers, and waterfalls in rural areas and the rural population [18]. The forest coverage rate (R3) reflects the availability of green resources in rural areas [19]. In the narrow sense, annual sunshine (R4) and annual precipitation (R5) reflect the abundance of light energy resources and the climate characteristics in rural areas, respectively [3].

### 3.4. Weight Method

The methods commonly used to determine weight are either subjective or objective. AHP is a typical subjective method; the entropy value method (EVM) is a typical objective method, but cannot assign different weights according to the importance of each index in theory. Commonly used methods of weight determination include subjective and objective methods. AHP is a weight-determination method that uses expert experience and existing knowledge to determine the importance of the index. The EVM is a weighting method calculated using survey data and determined according to the statistical properties of the index, but it cannot theoretically assign different weights according to the importance degree. Therefore, to combine subjective and objective index weighting, we combined the two weighting methods. In this study, AHP and EVM were used to jointly determine the weight of the index, satisfying the subjective and objective conditions [48] and the sum of the internal weight of each dimension is guaranteed to be 1. The subjective weight vector of indicators determined by AHP is defined as shown in Appendix A.

EVM is an objective weighting method. For example, entropy, criteria significance, and TOPSIS are the basis for evaluating measurable statistics. Although the outcomes may not be derived from analysis, the importance assigned to weights may vary [49]. The entropy-determined objective weighting vector can be described by the following steps:

(1) If an increase in the variable value leads to a worse situation, then

(3) $$z_{i}=\left(x_{\max}-x_{i}\right)/\left(x_{\max}-x_{\min}\right)$$

if an increase in the variable value leads to the best state of scenario, then

(4) $$z_{i}=\left(x_{i}-x_{\min}\right)/\left(x_{\max}-x_{\min}\right)$$

where x_i_ is the standardized value of an indicator for region i; the original values for region i are x_max_ and x_min_, which illustrate the region with the highest value and the lowest value, respectively.

(2) Since a logarithm was used in the entropy method, the normalized values cannot be used immediately. To copy the shadow caused by negative values, the sound value and translation value are normalized:

(5) $$Z_{i}=x_{i}+A$$

where Z_i_ is the value of translation and A is the magnitude of translation.

(3) By quantifying each index, the proportion of i region index under j index is calculated.

(6) $$p_{\mathrm{ij}}=Z_{i}/\sum Z\left(i=1,2\cdots \cdots ,n;j=1,2,\cdots \cdots ,m\right),$$

n is the number of regions and m is the number of indicators.

(4) Calculate the entropy value of j.

(7) $$e_{i}=-k/p_{\mathrm{ij}}\sum \ln\left(p_{\mathrm{ij}}\right),k=1/\ln\left(n\right),e_{j}>0.$$

(5) Calculate the coefficient of difference of j.

(8) $$g_{i}=1-e_{j}$$

(6) The coefficient of difference is normalized to calculate the weight of j.

(9) $$w_{i}=g_{i}/\sum g_{i}\left(j=1,2,\cdots ,m\right).$$

Weight coefficients within and between subsystems were obtained using the comprehensive weighting method, the AHP, the specific index system, and weight (Table 2). Table 2 displays the indicators coupled with their weights assigning results, which shows that the importance of the indicators is related to rural green development (GD). GD values are measured by indicators reflecting the most practical indicators in the field, and the overall impact of each indicator of rural green development on the positive or negative effect is demonstrated by these values [50,51]. Integrated weights are a combination of AHP weights and entropy weights. The higher the weight assigned, the greater the importance it reveals. For example, agricultural added value (0.0875) is the most important indicator in the response component. An increase in agricultural value-added will improve rural productivity and thus help improve the level of green rural development. 

## 4. Empirical Analysis 

### 4.1. Green Development Level Score and Degree Analysis

Firstly, we normalized the data of all indicators. To increase the final score, and thus improve the green development level, we applied an efficiency standardization treatment for indicators with larger values, and then performed a cost-oriented standardization treatment. Secondly, we used subjective and objective comprehensive weighting to determine the weight of each indicator. Finally, the weighted sum of the five dimensions was determined using the AHP to obtain the total score of green development (Formula (1)) in each region. According to the calculation results, the score of green development in rural areas in Shaanxi varied from 0.12 to 0.33 in 2018; the higher the score, the better the green development. With the green development scores of each region as the index, SPSS 13.0 statistical software was used to classify the advantages and disadvantages of green development for each province in China using the cluster analysis method. The cluster analysis showed that the green development of China can be divided into four categories: strong, medium, weak, and very weak. Areas with strong green development include Shenmu, Suide, and Huangling counties (Table 3). The regions with medium green development include Qishan, Luochuan, Lueyang, Hancheng, Foping, Taibai, and Fengxian, for a total of seven counties (Table 4). The weak areas include Zhouzhi, Meixian, Xingping, Liuba, Ningshan, and Fuxian, with a total of 22 counties (Table 5). Lantian, Yangxian, Mianxian Shiquan, and Jiaxian were very weak areas, with a total of 46 counties (Table 6).

**Figure 2:**
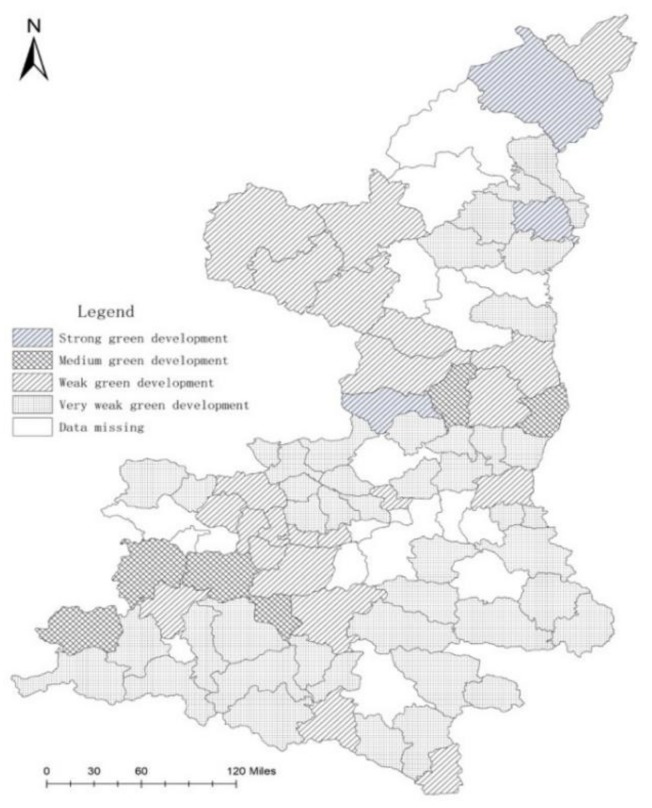
Rural green development degree of Shaanxi province.

The results showed that significant internal differences exist in the rural green development level in Shaanxi province, and green development is spatially agglomerated in regions, where the north is stronger, the southwest is moderate, the northwest is moderate, and the middle and the south are the worst (Figure 2).

Overall, Shaanxi province’s rural areas have few industrial structures in place, with a low level of social and economic development, low government management efficiency, insufficient rural green economic investment, and weak resident awareness of green development and other issues. For example, a high degree of development of the region will help optimize the industrial structure, increase environmental protection investment, and continue to increase the attention paid to green development by the local government. Government spending on environmental protection and investment in pollution control should be increased to promote coordination between the environment and economic development. Residents’ awareness of environmental protection should be raised, their quality of life and the ecological environment should be improved, land resources should be rationally planned and used, and the level of agricultural modernization should be enhanced. In areas with weak development, the construction of agricultural facilities should be increased, as should the use of resources, the structure of agricultural products should be improved and their competitiveness enhanced. Government policy and financial support need to be further improved. Where development is weak, the government should increase capital input, increase infrastructure construction, raise the level of economic development, increase the income of rural residents, and reduce the gap between urban and rural income. The evaluation can reflect the actual situation of rural green development in this province.

### 4.2. Analysis of Green Development Driving Type

**Figure 3:**
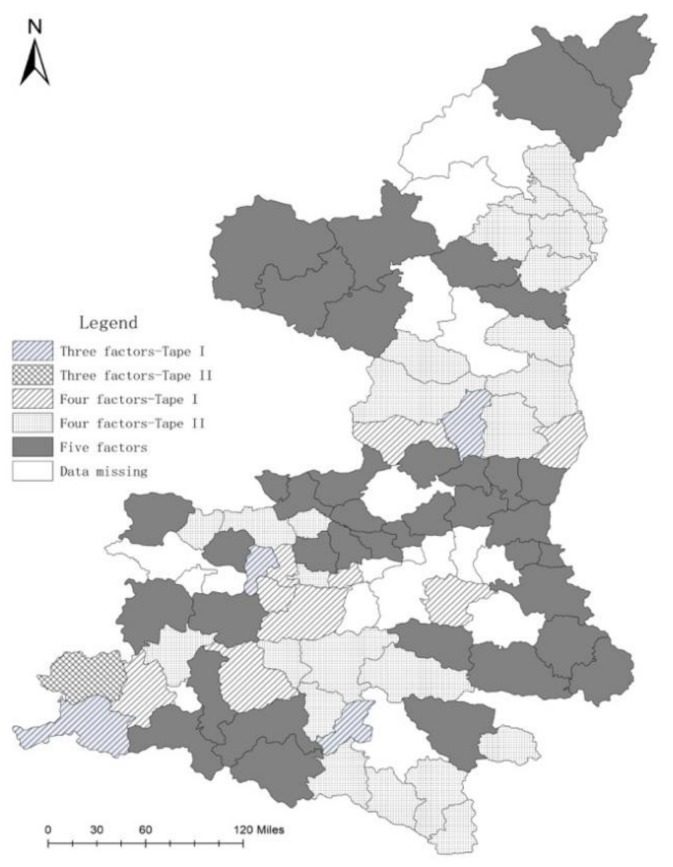
Spatial distribution of green development driving types in rural Shaanxi.

By analyzing the OECD model using the LSE method (Formula (2)), we identified five primary types of green development in rural Shaanxi based on drivers and causal mechanisms: Three-Factor Type I, Three-Factor Type II, Four-Factor Type I, Four-Factor Type II, and Five-Factor Type (Figure 3). We found that areas of the same type are adjacent to each other, as shown in Figure 3, which is known as spatial agglomeration. We leave the analysis of this relationship to future research.

#### 4.2.1. Three-Factor Type

In Three-Factor Type I (Table 7), government (G), the environment (E), and contribution to the quality of life (L) are the main factors, including Qishan, Ningqiang, Hanyin, and Luochuan counties. Qishan and Luochuan are areas with a medium green development level, whereas Ningqiang and Hanyin are areas with a weak development level. In areas with medium green development, the efficient use of resources is relatively high, environmental protection capacity is strong, and farmers’ living environment and sanitation conditions are of high quality. The government emphasizes rural green development in these areas and issued a number of policies and regulations to improve management efficiency. However, the second-level rural labor force transfer score is very low, which indicates that the employment level of agricultural production transfer to secondary and tertiary industries in these two areas is limited, and rural labor production capacity needs to be improved. Therefore, the government should continue to increase its investment in education, science, and technology to improve competition in the rural labor force. In terms of resource endowment, the two regions are different. In Luochuan, the per capita arable land area is the main problem because this area is located in the low-lying areas of the Loess plateau, with many mountains, deep gullies, and frequent natural disasters. However, the local water resources in Qishan are poor due to the excessive exploitation of water resources, which leads to serious ecological water use issues and the weak control of soil erosion. Therefore, while developing the economy, the government should plan and use resources reasonably. For Ningqiang and Hanyin, where the development level is very weak, environmental pressure performed relatively well for the three leading factors, with the contribution rate reaching 37.8% and 42.7% in the two regions, respectively. This indicates that these two regions have developed well in terms of agricultural production efficiency and rural sustainability. However, the added value of agriculture, forestry, animal husbandry, and fisheries in these regions is low. Overall, the green development levels in both regions are weak, and all indicators need to be strengthened.

Three-Factors Type II (G, E, and R) (Table 8) is dominated by three factors, the environment, government, and resource contribution effects, and only includes Lueyang, which is an area with a medium green development level. This area is rich in natural resources, with a forest area of 2.83 million km^2^ and a forest coverage rate of 45.2%, being listed as one of the province’s 23 forest districts. Within the territory are 10 rivers and a basin area of about 2014.6 km^2^. In addition, agricultural production in this area mainly focuses on the cultivation of Chinese medicinal herbs and the breeding of black chickens, which reduces the intensive use of pesticides, fertilizers, and plastic films, and the need for environmental protection. Therefore, its environmental contribution is relatively high. The government has strengthened its financial investment in science and technology, environmental protection, and urban construction to develop the region’s secondary and tertiary industries. However, neglect of agricultural and rural development has led to a low proportion of primary industry in GDP and low quality of life for rural residents. Therefore, improving the development of rural modernization would be conducive to enhancing the performance of the region and improving the level of rural green development.

#### 4.2.2. Four-Factor Type

Four-Factor Type I (E, L, S, and G) (Table 9) includes eight counties. Lantian and Zhouzhi, of these eight, were mainly affected by environmental pressure, quality of life, social economy, and government support. Huangling has a strong level of green development. Areas with a weak development level include Zhouzhi, Meixian, and Xingping. The development level is very weak for Lantian, Yang, and Mianxian. Regions with a strong level of development have a good background in agricultural development. Shaanxi is a base for high-quality apple production and export. The government not only vigorously developed the construction regulations for agricultural planting, but also provided considerable financial support for the improvement of rural science and technology, education, and residents’ quality of life. Resource endowment is not the driving factor of green development in this region; it is only a little weaker than the other factors but is still stronger than in other regions. The economic development degree of this region is higher given the better financial situation, and the government pays more attention to the environment in rural areas. Therefore, the quality of life of the residents in the region is better than in other regions. However, strengthening the management of soil erosion and arable land is still necessary. The per capita net income of rural residents, the number of agricultural machines, and the amount of agricultural plastic film are important factors in the development of the agricultural economy in the region, and significantly impact the improvement of rural economic development. However, affected by the Guanzhong economic development zone strategy, governments at all levels have formed a priority model for the development of secondary and tertiary industries. This development model increases pressure on the use of rural ecological resources, resulting in agricultural production being further developed. Although regions with a weak development degree have good local social and economic conditions, the high level of rural urbanization and the development of agricultural modernization and natural resources are insufficient due to their geographical location, which has become an important constraint on the green development of agriculture. Therefore, when developing the economy, the government should optimize the industrial structure, rationally plan and use ecological resources, and improve its management of natural resources.

The Four-Factor Type II (E, L, R, and G) (Table 10) is driven by environmental pressure, quality of life, resource endowment, and government support. Only two counties of this type, Suide and Foping, have a strong level of green development; 11 regions, such as Linyou and Wugong, have weak development and 13 regions, such as Qianyang and Yongshou, have very weak development. Suide has invested heavily in its workforce and has devoted many material and financial resources to rural administrative management and scientific and technological development, which has improved the green development efficiency of government management and the living standard of rural residents. Although water resources are abundant in the region, the water use efficiency is low, especially for agriculture. Therefore, water conservancy facilities need to be developed to improve water use efficiency for agriculture. The region simultaneously needs to optimize the agricultural production structure, improve the level of agricultural modernization, increase the income of rural residents, and narrow the urban-rural income gap. The county lags behind with regards to sewage, household waste treatment, and agricultural production mechanization, which increases environmental pressure to a certain extent. the ecological environment in areas with a moderate level of development remains somewhat less affected, so the environmental conditions are better than in other areas. Agricultural products in this region have a single structure, low added value, and low market competitiveness. Leading agricultural enterprises in line with local conditions need to be actively introduced, increases in agricultural added value need to be promoted, increasing green development. Natural resources in less-developed areas are in better condition, and the ecological environment is under less pressure. In most areas, the development of toilets and the per capita doctor occupancy rate in rural areas is relatively good, and the quality of life for rural residents is better than in other areas. The degree of agricultural mechanization per unit area in the region is relatively high, but it is concentrated in grain production, without an optimization trend. Therefore, the added value of agriculture is low and the level of agricultural modernization is not high. Development is very weak but, by contrast, the regional economic development level is limited, as is the government’s regulation and control ability. Rural production capacity and the level of science and technology can be improved. Agricultural production tends to prioritize planting rather than processing, with no obvious improvement in areas such as financial self-sufficiency and the income gap between urban and rural areas, which still need to be addressed via strengthened management.

Overall, for areas with weak or very weak green development, although the four aspects are the main driving factors, they have the same degree of relative inadequacy but are better than the absolute situation when including the fifth factor. In all these areas, despite the limited level of social and economic development, considerable room for improvement exists in terms of government management capacity, science and education level, per capita income of farmers, and efficiency in the use of natural resources. 

#### 4.2.3. Five Factors Type (E, L, G, R, and S) (Table 11)

The five factors of social economy, environmental pressure, quality of life, government support, and resource endowment have a common driving effect on green development in Shenmu with a strong degree of development; Taibai and Fengxian with a moderate degree; Fengxiang, Sanyuan, Dali, and Dingbian with a weak degree; and 28 regions (Table 5), including Yijun, Pucheng, and Shanyang, with a very weak degree, for 39 counties and districts in total (Table 6). Shenmu has the best green development in the rural areas of Shaanxi. It has a good local economic situation, a high level of financial self-sufficiency and urbanization, and the government has invested financial and material resources in rural development in science and technology, education, ecology, and the environment. Therefore, these five aspects are better than in other areas, and all factors have been included. The areas with a medium development level are the remote areas of the Qinling Mountains, which are rich in resources and have a suitable climate. However, restricted by topography, economic development is still dominated by traditional agriculture, resulting in a low level of economic development and a green development level hovering around the average. Transforming the model of economic development and improving the level of economic development are future goals. In areas with weak development, enterprises should actively promote resource-saving and environmentally friendly development modes when resources are scarce, economic development is relatively lagging, and government support is insufficient. In contrast, resources, economy, environment, and other aspects of regions with a weak development degree are all poor, and indicators under all dimensions need to be improved.

## 5. Conclusions

In this study, we selected 28 indicators based on the OECD LSE framework to assess green development in 78 rural areas of Shaanxi, considering local issues and limited data availability. Policies should focus on strengthening management plans to ensure the efficient use of available resources. Based on the LSE method, it can be better solved using quantitative analysis of regional differences in green development. Through the analysis of relevant numerical indicators, we revealed the spatial distribution of driving factors and types of green development in different regions and provided the necessary theoretical references for relevant departments to implement green agricultural development in accordance with local conditions. In this study, the contributions of OECD components in all research fields were carefully analyzed, and we found that environmental pressure and government support were the main factors influencing green development, followed by other restrictions. In these areas, increasing investment in environmental pollution control and strengthening policy intervention should be prioritized, followed by developing the socioeconomic capacity, improving the quality of life of villagers, and improving the use of natural resources. The OECD LSE results can be used to determine priorities in rural areas. Considering different weights and scenarios based on consultations with stakeholders and the acquisition of relevant information and experience will help future research focus on the assessment of green development to further assist policy-makers and planners when reviewing alternative programs and determining the potential impact of interventions in these areas of research.

Although we preliminarily evaluated and analyzed rural green development based on OECD-LSE, several problems remain to be studied. Firstly, our conclusions were based on county-level cross-sectional data from a single year (2018). If county-level panel data with a time cycle longer than 10 years or more are adopted, the internal differences and evolutionary trends of regional green development can be analyzed further [52]. Secondly, different index selection criteria and weight methods can produce different green development results, which affects the LSE analysis. In the future, a game theory could be used to study the interaction between the different variables to test the robustness and reliability of the existing analysis methods. Finally, the LSE approach is data-driven in nature, mainly through the statistical description of data, and lacks the theoretical basis of an explanatory model. Considering the impact of space, an exploratory spatial data analysis (ESDA) confirming the spatial data analysis method can further explain regional differences in green development [53]. 

## Figures and Tables

**Table 1:** Details of the indicators of rural green development in Shaanxi.

Dimensions	Variables	References and Data Sources
Social Economy	(%) Proportion of added value of primary production to GDP (+)	[22,47]
	(%) Urban and rural income ratio (−)	[3], [*]
	(CNY) Agricultural added value per 10,000 yuan (+)	[22,47]
	(%) Urbanization rate (+)	[22], [*]
	(%) Fiscal self-sufficiency rate (+)	[3,47]
	(%) Proportion of rural labor force transfer in population (+)	[21,47]
Environmental Pressure	(t/ha) Pesticide and fertilizer intensity (−)	[19,47]
	(t/ha) Intensity of agricultural plastic film use(−)	[19,47]
	(%) Sewage treatment rate (+)	[19], [*]
	(kw)Total power of agricultural machinery per unit area (+)	[3,47]
	(%) Harmless disposal rate of domestic garbage (+)	[15], [*]
Quality of Life	(%) Penetration rate of harmless toilets in rural areas (+)	[15], [*]
	(CNY) Per capita net income of rural residents (+)	[15], [*]
	(%) Air quality index (+)	[15], [*]
	(%) Per capita medical technician occupancy ratio (+)	[15], [*]
	(CNY) Total amount of consumer goods per capita (+)	[15,47]
	(t/ha) Grain yield (+)	[22], [**]
Government Support	(%) The number of villagers’ committees to the total population (+)	[8], [**]
	(%) Rural science and technology expenditure to public expenditure (+)	[22], [**]
	(%) Rural environmental protection expenditure to public expenditure (+)	[21], [**]
	(%) Water costs of rural agriculture, forestry to public expenditure (+)	[8], [**]
	Number of provincial agricultural leading enterprises (+)	[8], [*]
	(CNY) Per capita education expenditure (+)	[3], [**]
Resource Endowment	(ha) Rural per capita arable land area (+)	[3,47]
	(m^3^) Water resource per capita (+)	[19], [***]
	(%) Forest coverage rate (+)	[19], [***]
	(hour) Annual sunshine number (+)	[3], [***]
	(mm) Annual precipitation (+)	[3], [***]

Note: [*], [**], and [***] denote that the data source of indicators is from Shaanxi Provincial Bureau of Statistics, Municipal Statistics Bureau, and county-level statistical. bulletin of Shaanxi Province, respectively. (+), (−) represent that indicator is a positive value, a negative value correspondingly.

**Table 2:** Weights of dimensions and variables of 2018 Shaanxi’s rural green-development.

Dimensions	Variables	AHP	EVM	Integrated
Social economy (0.2)	Proportion of added value of primary production to GDP	0.0113	0.0998	0.0555
	Urban and rural income ratio	0.0138	0.0783	0.0461
	Agricultural added value per 10,000 yuan	0.0255	0.1496	0.0875
	Urbanization rate	0.0184	0.0808	0.0496
	Fiscal self-sufficiency rate	0.0705	0.3968	0.2337
	Proportion of rural labor force transfer in population	0.0099	0.1947	0.1023
Environmental pressure (0.2)	Pesticide and fertilizer intensity	0.0331	0.0793	0.0562
	Intensity of agricultural plastic film use	0.0130	0.1580	0.0855
	Sewage treatment rate	0.0251	0.0601	0.0426
	Total power of agricultural machinery per unit area	0.0089	0.6601	0.3344
	Harmless disposal rate of domestic garbage	0.0346	0.0427	0.0386
Life Quality (0.2)	Penetration rate of harmless toilets in rural areas	0.0336	0.1634	0.0985
	Per capita net income of rural residents	0.0173	0.3177	0.1675
	Air quality index	0.0136	0.1202	0.0669
	Per capita medical technician occupancy ratio	0.0136	0.2008	0.1072
	Total amount of consumer goods per capita	0.0095	0.0803	0.0449
	Grain yield	0.0089	0.1177	0.0633
Government support (0.2)	The number of villagers’ committees to total population	0.1218	0.12987	0.1258
	Rural science and technology expenditure to public expenditure	0.1757	0.35909	0.2674
	Rural environmental protection expenditure to public expenditure	0.0845	0.16445	0.1245
	The water costs of rural agriculture, forestry to public expenditure	0.0387	0.03545	0.0378
	Number of provincial agricultural leading enterprises	0.0414	0.23278	0.1371
	Per capita education expenditure	0.0627	0.07835	0.0705
Resource endowment (0.2)	Rural per capita arable land area	0.0219	0.23769	0.1298
	Annual sunshine number	0.0166	0.10291	0.0598
	Forest coverage rate	0.0153	0.01529	0.0153
	Annual precipitation	0.0251	0.04093	0.0330
	Water resource per capita	0.0359	0.60318	0.3195

**Table 3:** Regional score of green development level strong.

Areas	Score					
	S	E	L	R	G	Total
Shenmu	0.346	0.239	0.378	0.207	0.468	0.3274
Suide	0.060	0.223	0.182	0.757	0.252	0.2950
Huangling	0.281	0.430	0.311	0.133	0.255	0.2818

**Table 4:** Regional score of green development level medium.

Areas	Score					
	S	E	L	R	G	Total
Qishan	0.125	0.320	0.282	0.097	0.375	0.2400
Luochuan	0.106	0.476	0.265	0.120	0.225	0.2385
Lueyang	0.075	0.478	0.123	0.253	0.270	0.2397
Hancheng	0.290	0.329	0.372	0.112	0.229	0.2660
Fuoping	0.057	0.390	0.186	0.306	0.248	0.2373
Fengxian	0.139	0.261	0.264	0.177	0.266	0.2217
Taibai	0.155	0.286	0.124	0.331	0.330	0.2454

**Table 5:** Regional score of green development level weak.

Areas	Score					
	S	E	L	R	G	Total
Zhouzhi	0.130	0.314	0.300	0.083	0.145	0.1943
Meixian	0.106	0.318	0.254	0.095	0.178	0.1900
Xingping	0.131	0.300	0.304	0.078	0.106	0.1839
Linyou	0.101	0.209	0.143	0.228	0.367	0.2097
Fufeng	0.093	0.374	0.185	0.172	0.124	0.1900
Wugong	0.094	0.321	0.244	0.155	0.127	0.1885
Liuba	0.068	0.342	0.122	0.274	0.230	0.2071
Ningshan	0.064	0.415	0.146	0.186	0.174	0.1969
Ziyang	0.057	0.281	0.134	0.397	0.162	0.2064
Zhenpin	0.391	0.211	0.136	0.310	0.049	0.2195
Ganquan	0.091	0.273	0.236	0.123	0.211	0.1868
Fuxian	0.110	0.280	0.274	0.131	0.203	0.1994
Yichuan	0.088	0.241	0.250	0.139	0.251	0.2138
Fengxiang	0.150	0.301	0.229	0.102	0.191	0.1948
Sanyuan	0.140	0.277	0.286	0.098	0.159	0.1919
Dali	0.110	0.360	0.258	0.119	0.122	0.1921
Zhidan	0.297	0.224	0.214	0.159	0.177	0.2143
Wuqi	0.287	0.223	0.203	0.161	0.163	0.205
Fugu	0.266	0.261	0.273	0.181	0.191	0.2341
Jingbian	0.187	0.198	0.261	0.251	0.228	0.2252
Dingbian	0.171	0.221	0.225	0.373	0.156	0.2296

**Table 6:** Regional score of green development level very weak.

Areas	Score					
	S	E	L	R	G	Total
Ningqiang	0.083	0.313	0.157	0.083	0.193	0.1656
Hanyin	0.078	0.322	0.158	0.067	0.135	0.1521
Lantian	0.126	0.260	0.277	0.075	0.091	0.1657
Yangxian	0.097	0.265	0.164	0.074	0.156	0.1514
Mianxian	0.114	0.272	0.151	0.069	0.223	0.1662
Qianyang	0.057	0.269	0.112	0.123	0.214	0.1558
Yongshou	0.066	0.305	0.14	0.106	0.126	0.1491
Shiquan	0.069	0.317	0.18	0.099	0.136	0.1607
Langao	0.041	0.239	0.125	0.146	0.201	0.1507
Pingli	0.07	0.273	0.136	0.121	0.173	0.1549
Baihe	0.057	0.317	0.121	0.119	0.176	0.1584
Zhen’an	0.072	0.251	0.222	0.105	0.129	0.1564
Yanchang	0.079	0.271	0.16	0.181	0.207	0.1797
Mizhi	0.069	0.258	0.129	0.154	0.164	0.1553
Jiaxian	0.056	0.219	0.088	0.191	0.169	0.1451
Wupu	0.071	0.266	0.156	0.15	0.2161	0.1618
Qingjian	0.076	0.232	0.104	0.141	0.232	0.1574
Zizhou	0.063	0.205	0.119	0.125	0.209	0.1444
Yijun	0.111	0.233	0.171	0.139	0.22	0.1752
Longxian	0.094	0.25	0.123	0.129	0.122	0.1440
Jingyang	0.084	0.226	0.207	0.103	0.111	0.1470
Qianxian	0.112	0.259	0.238	0.104	0.105	0.1639
Liquan	0.151	0.224	0.274	0.089	0.12	0.1722
Binxian	0.189	0.22	0.214	0.099	0.1766	0.1803
Changwu	0.131	0.237	0.16	0.098	0.142	0.15398
Xunyi	0.104	0.182	0.143	0.116	0.141	0.1376
Chunhua	0.077	0.257	0.131	0.144	0.141	0.1501
Tongguan	0.092	0.345	0.176	0.107	0.138	0.1719
Heyang	0.08	0.243	0.128	0.146	0.114	0.1427
Chengcheng	0.082	0.274	0.139	0.141	0.125	0.1525
Pucheng	0.113	0.259	0.156	0.12	0.178	0.1657
Baishui	0.093	0.3	0.162	0.12	0.228	0.1811
Fuping	0.108	0.243	0.183	0.117	0.118	0.1543
Huayin	0.092	0.254	0.173	0.09	0.077	0.1375
Nanzheng	0.135	0.267	0.155	0.088	0.159	0.1614
Chenggu	0.141	0.314	0.17	0.086	0.13	0.1687
Xixiang	0.097	0.351	0.158	0.093	0.152	0.1710
Zhenba	0.073	0.225	0.071	0.119	0.113	0.1205
Xunyang	0.091	0.295	0.135	0.101	0.18	0.1607
Luonan	0.097	0.236	0.136	0.103	0.093	0.1334
Danfeng	0.072	0.233	0.118	0.086	0.133	0.1287
Shangnan	0.085	0.253	0.074	0.112	0.179	0.1412
Shanyang	0.074	0.233	0.132	0.105	0.179	0.1434
Zhashui	0.141	0.258	0.112	0.139	0.233	0.1454
Yanchuan	0.131	0.213	0.153	0.141	0.185	0.1768
Zichang	0.057	0.269	0.151	0.069	0.223	0.1651

**Table 7:** Contribution rate of green development driving effect of “Three-Factors Type I” areas.

Driving Type	Areas	Level	S	E	L	R	G
Three-Factors Type I	Qishan	(3)	10.4%	26.7%	23.5%	8.1%	31.3%
	Ningqiang	(1)	10%	37.8%	18.9%	10%	23.3%
	Hanyin	(1)	10.2%	42.4%	20.7%	8.9%	17.7%
	Luochuan	(3)	10.6%	47.6%	26.5%	12%	22.5%

Note: (1), (2), (3), (4) stand for the level of green development, as follows: (1): very weak (2): weak (3): Medium (4): Strong.

**Table 8:** Contribution rate of green development driving effect of “Three Factors Type II” areas.

Driving Type	Areas	Level	S	E	L	R	G
Three-Factors Type II	Lueyang	(3)	6.2%	39.9%	10.3%	21.1%	22.5%

Note: (1), (2), (3), (4) stand for the level of green development, as follows: (1): very weak (2): weak (3): Medium (4): Strong.

**Table 9:** Contribution rate of green development driving effect of “Four-Factors Type I” areas.

Driving Type	Areas	Level	S	E	L	R	G
Four-Factors Type I	Lantian	(1)	15.2%	31.2%	33.6%	9.1%	10.9%
	Zhouzhi	(2)	13.3%	32.4%	31%	8.5%	14.9%
	Meixian	(2)	11.1%	33.4%	26.7%	10%	18.8%
	Xingping	(2)	14.2%	32.6%	33.1%	8.6%	11.6%
	Hancheng	(3)	21.7%	24.7%	28%	8.2%	17.2%
	Yangxian	(1)	12.8%	35%	21.7%	9.8%	20.6%
	Mianxian	(1)	13.7%	32.8%	18.2%	8.4%	26.9%
	Huangling	(4)	19.9%	30.5%	22%	9.4%	18.1%

Note: (1), (2), (3), (4) stand for the level of green development, as follows: (1): very weak (2): weak (3): Medium (4): Strong.

**Table 10:** Contribution rate of green development driving effect of “Four-Factors Type II” areas.

Driving Type	Areas	Level	S	E	L	R	G
Four-Factors Type II	Qianyang	(1)	7.4%	34.6%	14.5%	15.9%	27.6%
	Linyou	(2)	9.6%	20%	13.6%	21.7%	35.1%
	Fufeng	(2)	9.8%	39.4%	19.5%	18.1%	13%
	Yongshou	(1)	8.8%	41%	18.8%	14.3%	17%
	Wugong	(2)	9.9%	34%	26%	16.5%	13.5%
	Liuba	(2)	6.5%	33%	11.8%	26.5%	22.2%
	Fuoping	(3)	4.8%	32.8%	15.7%	25.8%	20.9%
	Shiquan	(1)	8.6%	39.5%	22.5%	12.3%	17%
	Ningshan	(2)	6.5%	42.1%	14.9%	18.9%	17.7%
	Ziyang	(2)	5.6%	27.2%	13%	38.5%	15.7%
	Langao	(1)	5.4%	31.8%	16.7%	19.4%	26.6%
	Pingli	(1)	9%	35.4%	17.6%	15.6%	22.4%
	Zhenpin	(2)	4.5%	28.2%	12.4%	19.3%	35.6%
	Baihe	(1)	7.2%	40.1%	15.3%	15.1%	22.3%
	Zhen’an	(1)	9.3%	32.1%	28.5%	13.5%	16.6%
	Yanchang	(1)	8.8%	30.1%	17.8%	20.1%	23%
	Ganquan	(2)	9.7%	29.2%	25.3%	13.2%	22.6%
	Fuxian	(2)	10.9%	28%	27.4%	13.1%	20.3%
	Yichuan	(2)	8.8%	34%	25%	14%	25.1%
	Huanglong	(2)	7.4%	26.9%	23.3%	24%	18.4%
	Suide	(4)	4%	15.1%	12.3%	51.4%	17.1%
	Mizhi	(1)	9%	33.4%	16.7%	19.8%	21.2%
	Jiaxian	(1)	7.8%	30.3%	12.2%	26.4%	23.3%
	Wupu	(1)	8.9%	33%	19.3%	18.6%	20.2%
	Qingjian	(1)	9.7%	29.6%	13.3%	17.9%	29.5%
	Zizhou	(1)	8.7%	28.4%	16.5%	17.3%	29%

Note: (1), (2), (3), (4) stand for the level of green development, as follows: (1): very weak (2): weak (3): Medium (4): Strong.

**Table 11:** Contribution rate of green development driving effect of “Five-Factors Type” areas.

Driving Type	Areas	Level	S	E	L	R	G
Five-factors Type	Yijun	(1)	12.7%	26.6%	19.6%	19.6%	25.1%
	Fengxiang	(2)	15.4%	31%	23.6%	10.5%	19.6%
	Longxian	(1)	13.1%	34.8%	17.1%	18%	17%
	Fengxian	(3)	12.5%	23.6%	23.8%	16%	24.1%
	Taibai	(3)	12.7%	23.4%	10.1%	27%	26.9%
	Sanyuan	(2)	14.5%	28.9%	29.8%	10.2%	16.6%
	Jingyang	(1)	11.5%	30.9%	28.2%	14.1%	15.2%
	Qianxian	(1)	13.7%	31.7%	29.1%	12.7%	12.8%
	Liquan	(1)	17.6%	26%	31.9%	10.4%	14%
	Binxian	(1)	21.1%	24.5%	23.8%	11.1%	19.6%
	Changwu	(1)	17%	30.8%	20.9%	12.7%	18.5%
	Xunyi	(1)	15.1%	26.6%	20.9%	16.9%	20.5%
	Chunhua	(1)	10.4%	34.3%	17.4%	19.2%	18.7%
	Tongguan	(1)	10.7%	40.1%	20.5%	12.5%	16.1%
	Dali	(2)	11.4%	37.5%	26.9%	12.4%	11.7%
	Heyang	(1)	11.2%	34.2%	18%	20.5%	16.1%
	Chengcheng	(1)	10.8%	36%	18.3%	18.5%	16.4%
	Pucheng	(1)	13.7%	31.4%	18.9%	14.6%	21.5%
	Baishui	(1)	10.3%	33.2%	17.9%	13.3%	25.3%
	Fuping	(1)	14%	31.6%	23.8%	15.2%	15.4%
	Huayin	(1)	13.4%	37%	25.2%	13.2%	11.3%
	Nanzheng	(1)	16.8%	33.1%	19.3%	11%	19.7%
	Chenggu	(1)	16.8%	37.3%	20.3%	10.3%	15.4%
	Xixiang	(1)	11.4%	41.1%	18.6%	11%	17.9%
	Zhenba	(1)	12.2%	37.4%	11.9%	19.8%	18.8%
	Xunyang	(1)	11.4%	36.7%	16.8%	12.6%	22.4%
	Luonan	(1)	14.6%	35.5%	20.4%	15.5%	14%
	Danfeng	(1)	11.2%	36.3%	18.5%	13.4%	20.7%
	Shangnan	(1)	12.1%	35.9%	10.6%	15.9%	25.4%
	Shanyang	(1)	13.9%	35.8%	21.1%	10.7%	18.5%
	Zhashui	(1)	10.3%	32.2%	18.3%	14.5%	24.7%
	Yanchuan	(1)	15.9%	29.2%	12.7%	15.8%	26.4%
	Zichang	(1)	15.9%	25.8%	18.6%	17.2%	22.5%
	Zhidan	(2)	27.8%	20.9%	20%	14.8%	16.5%
	Wuqi	(2)	26.6%	20.6%	18.8%	14.9%	19%
	Shenmu	(4)	21.1%	14.6%	23.1%	12.6%	28.6%
	Fugu	(2)	22.7%	22.3%	23.3%	15.4%	16.3%
	Jingbian	(2)	20.2%	22.3%	23.2%	17.6%	16.6%
	Dingbian	(2)	15%	19.3%	19.6%	32.5%	13.6%

Note: (1), (2), (3), (4) stand for the level of green development, as follows: (1): very weak (2): weak (3): Medium (4): Strong.

## Appendix A

Important aspects of the AHP weights of each index in this study are composed of experience judgment and expert consultation. While evaluating AHP, Table A1, Table A2, Table A3, Table A4 and Table A5 demonstrate the comparison matrix of urban and rural areas with similar weights of each index. Experts are willing to keep their personal information as private.

**Table A1:** Pairwise comparison matrix of Social Economy.

	S1	S2	S3	S4	S5	S6	Weight
S1	1	1/2	1	1/3	1/4	1	0.0113
S2		1	1/3	1	1/5	1	0.0138
S3			1	3	1/5	3	0.0255
S4				1	1/4	3	0.0184
S5					1	6	0.0705
S6						1	0.0099

**Table A2:** Pairwise comparison matrix of Environmental Pressure.

	E1	E2	E3	E4	E5	Weight
E1	1	1	3	4	1	0.0331
E2		1	1/3	1	1/3	0.0130
E3			1	3	1	0.0251
E4				1	1/5	0.0089
E5					1	0.0346

**Table A3:** Pairwise comparison matrix of Quality of Life.

	L1	L2	L3	L4	L5	L6	Weight
L1	1	3	3	3	2	3	0.0336
L2		1	1	3	3	1	0.0173
L3			1	1	1	2	0.0136
L4				1	2	3	0.0136
L5					1	1	0.0095
L6						1	0.0089

**Table A4:** Pairwise comparison matrix of Government Support.

	G1	G2	G3	G4	G5	G6	Weight
G1	1	1/3	3	2	3	3	0.1218
G2		1	3	3	3	2	0.1757
G3			1	3	2	3	0.0845
G4				1	1	1/3	0.0387
G5					1	1/2	0.0414
G6						1	0.0627

**Table A5:** Pairwise comparison matrix of Resource Endowment.

	R1	R2	R3	R4	R5	Weight
R1	1	2	3	1/2	1/3	0.0219
R2		1	1	1	1/2	0.0166
R3			1	1	1/2	0.0153
R4				1	1	0.0251
R5					1	0.0359
